# Recent advances in understanding the effects of lignin structural characteristics on enzymatic hydrolysis

**DOI:** 10.1186/s13068-021-02054-1

**Published:** 2021-10-20

**Authors:** Yufeng Yuan, Bo Jiang, Hui Chen, Wenjuan Wu, Shufang Wu, Yongcan Jin, Huining Xiao

**Affiliations:** 1 Jiangsu Co-Innovation Center of Efficient Processing and Utilization of Forest Resources, Nanjing Forestry University, Nanjing, 210037 China; 2 Department of Chemical Engineering, University of New Brunswick, Fredericton, NB E3B 11 5A3 Canada; 3 Laboratory of Wood Chemistry, Nanjing Forestry University, 159 Longpan Rd, Nanjing, 210037 China

**Keywords:** Lignocellulose, Enzymatic hydrolysis, Lignin, Cellulase, Interaction

## Abstract

Enzymatic hydrolysis of lignocellulose for bioethanol production shows a great potential to remit the rapid consumption of fossil fuels, given the fact that lignocellulose feedstocks are abundant, cost-efficient, and renewable. Lignin results in low enzymatic saccharification by forming the steric hindrance, non-productive adsorption of cellulase onto lignin, and deactivating the cellulase. In general, the non-productive binding of cellulase on lignin is widely known as the major cause for inhibiting the enzymatic hydrolysis. Pretreatment is an effective way to remove lignin and improve the enzymatic digestibility of lignocellulose. Along with removing lignin, the pretreatment can modify the lignin structure, which significantly affects the non-productive adsorption of cellulase onto lignin. To relieve the inhibitory effect of lignin on enzymatic hydrolysis, enormous efforts have been made to elucidate the correlation of lignin structure with lignin–enzyme interactions but with different views. In addition, contrary to the traditional belief that lignin inhibits enzymatic hydrolysis, in recent years, the addition of water-soluble lignin such as lignosulfonate or low molecular-weight lignin exerts a positive effect on enzymatic hydrolysis, which gives a new insight into the lignin–enzyme interactions. For throwing light on their structure–interaction relationship during enzymatic hydrolysis, the effect of residual lignin in substrate and introduced lignin in hydrolysate on enzymatic hydrolysis are critically reviewed, aiming at realizing the targeted regulation of lignin structure for improving the saccharification of lignocellulose. The review is also focused on exploring the lignin–enzyme interactions to mitigate the negative impact of lignin and reducing the cost of enzymatic hydrolysis of lignocellulose.

## Background

**Fig. 1:**
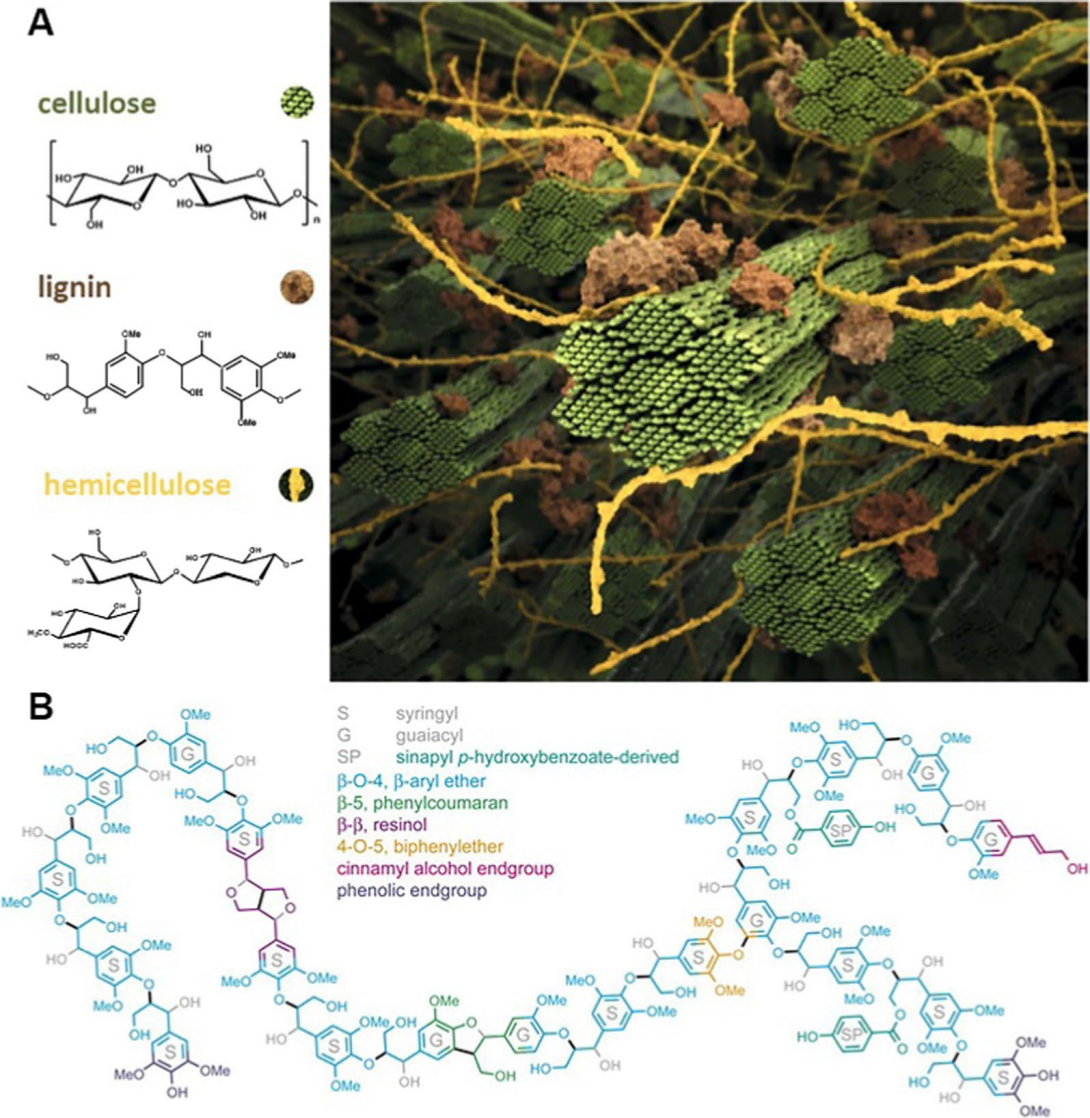
Structure of lignocellulosic biomass and its components (**A**) [18] and representation of a lignin polymer from poplar, as predicted from NMR-based lignin analysis (**B**) [19]

To reduce the negative impact of fossil fuels on energy and the environment, lignocellulose, as an abundant, green and renewable resource, has been widely used for bioethanol production to replace fossil fuels nowadays. Lignocellulose is rich in cellulose (40–50%) along with hemicelluloses (20–30%) and lignin (15–30%) [1]. The linear cellulose and branched hemicelluloses are the main polysaccharides for green biofuels production via biorefinery technology [2, 3]. However, the presence of lignin in the cell walls confers rigidity to the lignocellulose, which prevents the structural polysaccharides from degradation by microorganisms and enzymes (Fig. 1A). Lignin is an aromatic polymer with a three-dimensional network structure derived from three basic phenylpropanolic monomers, i.e., *p*-coumaryl, coniferyl, and sinapyl alcohols [4, 5]. The three monolignols are incorporated into lignin biomacromolecule with the units of guaiacyl (G), syringyl (S) and *p*-hydroxyphenyl (H) and linkages of β-*O*-4, β–β, α-*O*-4, 4-*O*-5, β-5, etc. (Fig. 1B).

In the process of enzymatic saccharification of lignocellulose, the non-productive adsorption of enzyme onto lignin caused by the hydrogen bonding, hydrophobic, and electrostatic interactions are thought to be the main reasons for the low saccharification efficiency of lignocellulose [6]. Steric hindrance of lignin macromolecule and lignin-based derivatives produced during the pretreatment process also shows negative effects on subsequent enzymatic hydrolysis. In addition, lignin is prone to co-precipitate with the enzyme protein, resulting in the irreversible adsorption of enzyme protein onto lignin. The protein folded structure is lost during the adsorption, thus decreasing the enzymatic activity [7, 8]. To reduce or minimize the inhibitory effect of lignin, a number of measures have been employed, including the pretreatment, the use of additives and the genetic engineering of lignin biosynthesis. Pretreatment that removes part of the lignin and increases the accessible surface area of the substrate to cellulase is the most critical step to enhance the enzymatic saccharification efficiency [9]. The lignin-blocking additives (Tween 80, bovine serum protein (BSA), polyethylene glycol (PEG) and the metal ions, etc.) have also been reported that can decrease the non-productive adsorption of cellulase onto lignin. For example, Tween 80 and BSA could mitigate the unproductive adsorption of cellulase on lignin by binding with lignin, thus improving the access of enzymes to cellulose [10, 11]. Lin et al. [12] found that the addition of PEG could reduce the non-productive adsorption of cellulase onto lignin by forming the PEG–cellulase complex, which could disperse cellulase and avoid cellulase aggregation. Akimkulova et al. [13] suggested that Mg^2+^ weaken the non-productive adsorption of cellulase onto lignin driven by the electrostatic interaction and hydrogen-bonding. In addition, genetic manipulation of plants can also reduce the non-productive adsorption of cellulase onto lignin by reducing the lignin content or regulating the lignin biosynthesis [14, 15].

In recent years, several novel studies have found that water-soluble lignin (WSL), such as micromolecular lignin and lignosulfonate (LS), can promote enzymatic hydrolysis [16, 17], which gives a new insight in improving the enzymatic hydrolysis efficiency. However, the mechanism of enzymatic hydrolysis promoted by WSL is still unclear. At present, understanding the interaction between lignin and cellulase has become an important means to investigate the influence of lignin on enzymatic hydrolysis. The relationship of lignin with enzymatic hydrolysis efficiency, and the mechanism of interaction between lignin and cellulase are still worth illustrating further. Based on these, the recent progress on the effects of lignin content, distribution, structure and source on enzymatic hydrolysis, as well as the mechanism of lignin–cellulase interaction is critically reviewed in this article. Furthermore, this review also highlights the future perspective of the research focusing on the influence of lignin on enzyme protein activity by exploring the interactions between these two biomolecules.

## Effect of residual lignin on enzymatic hydrolysis

### Content of residual lignin in substrate

The presence of lignin largely hinders the accessibility of enzymes to cellulose, which results in a very low enzymatic hydrolysis of the lignocellulose feedstock [20, 21]. Pretreatment is a key step in improving the efficiency of enzymatic hydrolysis by removing lignin and reducing the recalcitrance of lignocellulose [22–24]. Currently, the most important pretreatment methods for reducing the lignin content include the alkali, sulfite, organosolv, ionic liquids, and deep eutectic solvents pretreatments. The removal of lignin could significantly accelerate the enzymatic hydrolysis of lignocellulose. Alkali pretreatment, such as NaOH, Na_2_CO_3_ and green liquor (GL), has been widely used to improve the digestibility of cellulose by removing the lignin. The ether linkages in lignin could be readily cleaved under alkaline and high-temperature conditions, resulting in an increased delignification, thus improving the accessibility of cellulase to cellulose. Sulfite pretreatment, including the sodium sulfite pretreatment and ammonium sulfite pretreatment, can also effectively remove lignin. During the sulfite pretreatment, the sulfite groups can attack the aliphatic side chains of lignin and replace the hydroxyl and/or ether functional groups. Consequently, lignin was largely sulfonated and became more hydrophilic. This change reduced the non-productive adsorption of cellulase onto lignin and steric hindrance [25, 26]. Excepting some organosolv pretreatments carried out under acidic conditions (Park et al. [27] showed that sulfuric acid–ethanol or magnesium chloride–ethanol rarely reduced lignin content of pitch pine. The original lignin content of pitch pine was 26.16%. After treating by sulfuric acid–ethanol or magnesium chloride–ethanol, it was merely decreased to 23.78% and 25.78%), most organosolv pretreatment also leads to a distinct improvement of enzymatic hydrolysis efficiency by extracting lignin from lignocellulosic materials, which also exposes more accessible surface area of cellulose and improves the enzymatic hydrolysis efficiency[28]. It is generally believed that the dissolution of lignin by ionic liquids is achieved by the basicity of the hydrogen bonds of anions and the acidity of the hydrogen bonds of cations, the π–π interactions and hydrophobic interactions [29]. Deep eutectic solvent is a transparent mixture with a low freezing point obtained by the interaction between the hydrogen bond donor and the hydrogen bond acceptor [30]. The mechanism of deep eutectic solvent dissolving lignin is similar to that of ionic liquids, but it is more selective for extracting lignin rather than cellulose. Several common pretreatment methods for lignin removal and their effects on enzymatic hydrolysis are summarized in Table 1. Brienzo et al. [31] also investigated the enzymatic hydrolysis rate of sugarcane bagasse with different lignin content, and found that the samples with low lignin content generally showed a high glucose yield. Zhang et al. [32] used a novel film model composed of only lignin and cellulose to investigate the effect of lignin content on enzymatic hydrolysis. The results noticed that low lignin content in the film was beneficial for improving enzymatic hydrolysis rate. Hao et al. [33] prepared two lignin-rich residues (LRR-DA and LRR-NaOH) from corn stover through dilute acid (DA) pretreatment and dilute alkali (NaOH) pretreatment. The LRR-DA exhibited a stronger inhibitory effect on enzymatic hydrolysis than LRR NaOH. This may be due to the higher surface lignin content of LRR-DA. Zhou et al.[34] found that the glucose yield of green liquor (GL)–sulfite pretreated sugarcane bagasse continued to increase within 72 h, which was related to the low lignin content in the sugarcane bagasse. The above cases indicated that lignin content played a key role in the enzymatic hydrolysis and lignin content was negatively correlated with enzymatic hydrolysis efficiency.

**Table 1:** Several common pretreatment methods for lignin removal and their effects on enzymatic hydrolysis

Substrate	Pretreatment	Enzymatic hydrolysis	Lignin removal/%	Result	Refs.
Wheat straw	2% NaOH, 80 °C, and 2 h	10%, 150 rpm, 50 °C, 72 h, and pH = 4.8	71.8	Increasing the hydrolysis yield by 32.4%	[39]
Corn stover	NaOH + Na_2_S, 8% total titratable alkali, 40% sulfidity, 140 °C, and 1 h	5%, 180 rpm, 50 °C, 48 h, and pH = 4.8	45.0	The original polysaccharides conversion: 70.0%	[40]
Barley straw	2% NaOH, 105 °C, and 10 min	1%, 180 rpm, 50 °C, and pH = 4.8	84.8	The reducing sugar yield: 86.5%	[41]
Corncob	12% sodium sulfite, 160 °C, 20 min and pH = 7	2%, 95 rpm, 50 °C, 48 h, and pH = 4.8	77.4	Increasing the glucose yield by 20.1%	[25]
Wheat straw	20% ammonium sulfite, 4% sodium carbonate, 180 °C, and 1 h	2%, 50 °C, 24 h, and pH = 5.0	82.6	99.9% of glucan and 88.0% of xylan were hydrolyzed	[26]
Eucalyptus	50% 2-propanol, 220 °C, and 2 h	5%, 150 rpm, 50 °C, 72 h, and pH = 4.8	81.3	The glucose yields: 88.6%	[42]
Wheat straw	70% Glycerol, 220 °C, and 3 h	2%, 150 rpm, 50 °C, 24 h, and pH = 4.8	65.0	The reducing sugar conversion: 90.0%	[43]
Eucalyptus	80% tetrahydro-2-furanmethanol, 1% HCl, 120–180 °C, and 30 min	2%, 150 rpm, 50 °C, 72 h, and pH = 4.8	94.0	The hydrolysis conversion: 94.2%	[44]
Corncob	Choline chloride: Glycerol (1:2), 90 °C, and 24 h	0.25%, 180 rpm, 50 °C, 72 h, and pH = 4.8	87.6	The glucose yield: 85.3%	[32]
Eucalyptus	Choline chloride: lactic acid (1:10), 110 °C, and 6 h	2%, 150 rpm, 50 °C, 72 h, and pH = 4.8	80.0	The glucose yield: 94.3%	[45]
Corncob	Choline chloride: imidazol (3:7), 150 °C, and 15 h	6%, 180 rpm, 50 °C, 80 h, and pH = 4.8	88.0	The glucose yield: 94.6%	[46]
Corn stalk	*N*-methyl-2-pyrrolidonium chloride/H_2_O, 90 °C, and 30 min	1%, 150 rpm, 50 °C, 72 h, and pH = 4.8	85.9	The glucose yield: 79.6%	[47]
Switchgrass	1-Ethyl-3-methylimidazolium acetate,160 °C, and 3 h	0.5%, 150 rpm, 50 °C, 24 h, and pH = 4.8	69.2	The glucose yield: 96.0%	[48]
Rice straw	1-Buthyl-3-methylimidazolium acetate, 120 °C, and 5 h	3%, 100 rpm, 45 °C, 72 h, and pH = 4.8	41.4	The glucose yield: 95.9%	[49]

In addition, genetic engineering to reduce the content of lignin has been shown to increase the efficiency of enzymatic hydrolysis. The rigid and compact structure of plant cell walls contributes to the lignocellulose recalcitrance to enzymatic hydrolysis. The chemical compositions of biomass, which form a protective barrier to block the access of enzyme to substrate, are major causes of the recalcitrance of biomass. Among the chemical compositions of biomass, lignin is the most important component of cell wall recalcitrance to saccharification, especially in the process of enzymatic hydrolysis. Chen and Dixon [35] suggested that the recalcitrance of transgenic alfalfa to acid pretreatment and enzymatic hydrolysis was found to be proportional to the lignin content. Edmunds et al. [36] found that glucose release of transgenic *Pinus taeda* could increase 1.9- to 3.2-fold due to the reduction of lignin compared to the control. Davison et al. [37] found that the xylose yield of acid-hydrolyzed poplar genetic varieties with low lignin content and S/G ratio was 25% higher than those with high lignin content and S/G ratio. Mansfied et al.[38] manipulated lignin content to assess the effect of cell wall lignin on hydrolysis efficiency, and found that low-lignin plants showed up to a 15% increase in conversion rate, which was close to complete hydrolysis of cellulose polymers.

Although removing lignin can improve the efficiency of lignocellulose enzymatic hydrolysis, the complete removal of lignin is not necessary. This is because when the lignin removal rate reaches a certain level, the promotion effect of lignin removal on the enzymatic hydrolysis of the pretreated substrate gradually weakens. Zhang et al.[32] used DES (lactic acid and choline chloride) to treat the corncob at 90 °C for 24 h and found that once the delignification was over 70%, the further removal of lignin would not promote the yield of reducing sugar. Moreover, the pretreatment process, especially those harsh methods that use strong acid or base, not only promote the delignification of biomass, but also facilitate the degradation of carbohydrates. In general, the pretreatment process that leads to a high lignin removal rate also results in a substantial sugar loss. Therefore, the promotion effect of high lignin removal rate on enzymatic saccharification may be neutralized by the sugar loss during the pretreatment process. It is necessary to find a balance between the removal of lignin and the loss of carbohydrate when designing a suitable pretreatment method for the enzymatic hydrolysis of lignocellulosic biomass [39]. In addition, excessive lignin removal results in high costs associated with processing, high chemical recovery cost, and poor environmental benefit. Therefore, future studies should be focused on optimizing pretreatment parameters under mild pretreatment conditions and recycling the chemical reagents to achieve high enzymatic hydrolysis efficiency with low capital and operation costs.

### Distribution of residual lignin in substrate

In addition to lignin content, the deposition and redistribution of lignin are considered as crucial factors to determine the cell wall recalcitrance to enzymatic hydrolysis [50, 51]. It has been reported that hydrothermal, ammonia fiber expansion (AFEX), steam explosion and dilute sulfuric acid pretreatment led to the migration and redistribution of lignin, which causes the structure changes of the lignocellulose matrix. Hydrothermal pretreatment induces the migration of lignin and results in the opening and dilation of pore volume and specific surface area, which facilitates enzymatic hydrolysis [52]. Wang et al. [53] observed that hydrothermal pretreatment effectively improved 3.5-fold fermentable sugars conversion compared with untreated biomass by removing hemicellulose and migrating lignin at 190 °C for 15 min. AFEX pretreatment can disrupt the linkages in lignin–carbohydrate complex (LCC), partially deconstruct the hemicellulose and lignin and migrate lignin to the cell wall surface, resulting in the formation of porous structures. The porous structure further improved enzymatic hydrolysis efficiency [54]. Abdul et al. [55] observed that AFEX pretreatment could promote the enzymatic hydrolysis of oil palm empty fruit bunch. The removal and redistribution of lignin caused by AFEX pretreatment increased the accessibility of enzyme to cellulose. The liquid ammonia pretreatment also has been reported to result in the lignin relocation, which improves the enzyme access to cellulose [56]. Furthermore, Zhu et al. [57] found that ethylenediamine pretreatment exposed a more accessible surface of the substrate by the coalescence and relocalization of the lignin, thus enhancing the productive adsorption of cellulase to cellulose. In addition to the above pretreatment methods, steam explosion pretreatment also resulted in the improvement of enzymatic hydrolysis rate due to increased pore volume and specific surface area of the substrate caused by the redistribution and removal of lignin [58].

The condensation of lignin and the formation of pseudo-lignin have been reported to inhibit enzymatic hydrolysis. For example, the formation of spherical lignin droplets in the hydrothermal pretreatment shows the inhibitory effect on enzymatic hydrolysis [59]. Zhao et al. [60] reported that AFEX pretreatment at 2.0 g/g (H_2_O_2_/substrate) loading even increased the lignin content of moso bamboo compared to the raw material. This was due to the recondensation of lignin droplets to form new macromolecular lignin and the degradation of hemicellulose to form the pseudo-lignin. Similar observation also indicates that for the liquid hot water pretreatment, the hydrolysis rate of wheat straw gradually decreases over time due to the accumulation of lignin droplets on the surface of the hydrolyzed substrate [61]. Shinde et al. [62] reported that the formation of lignin-like polymer termed pseudo-lignin occurred in dilute acid pretreatment. Similar to residual lignin, pseudo-lignin plays a negative role in enzymatic hydrolysis because of its unproductive adsorption to the enzymes. For example, He et al. [63] observed that pseudo-lignin was formed by dehydration and aromatization of carbohydrates during dilute acid pretreatment, which absorbed the enzyme and reduced the efficiency of enzymatic hydrolysis. Hu et al. [64] also found that compared to a 50/50 mixture of pseudo-lignin and dilute acid pretreated lignin, pseudo-lignin had a greater inhibition impact on enzymatic hydrolysis, which was attributed to its high hydrophobicity induced by the methoxy and polyaromatic structures presented in pseudo-lignin. The redistribution of lignin can disrupt the compact structure of lignocellulose and result in the exposure of cellulose on enzyme, but lignin itself still remains harmful to enzymatic hydrolysis.

### Structural of residual lignin in substrate

The S/G ratio and relative abundance of the three monomers of lignin have been found to have an impact on the enzymatic hydrolysis rate. Studer et al. [65] found that the high S/G ratio would be beneficial to the release of glucose and xylose of pretreated samples. The enzymatic hydrolysis rate of corn stover was much higher than that of switchgrass under the milder conditions of pretreatment and similar enzyme dosage [66]. Further studies found that the S/G ratio of lignin in corn stover was 1.4 and 0.8 in switchgrass [67–69], which was consistent with the finding that the high S/G was related to less pretreatment severity and inhibition effect on enzymatic hydrolysis [70]. However, inconsistent results have also been reported. Xu et al. [71] found that the lignin with the high S/G ratio enhanced the non-productive adsorption of cellulase and was not conducive to the enzymatic saccharification of cellulose. Tan et al. [72] observed that a high S/G ratio of bisulfite pretreated oil palm empty fruit bunch lignin was negatively correlated with the enzymatic hydrolysis efficiency. Pape et al. [70] indicated that the S/G ratio of Eucalyptus wood had little effect on the saccharification efficiency of ionic liquid pretreated biomass.

For untreated biomass, Pape et al. [70] showed that a high S/G ratio produced a lower enzymatic hydrolysis efficiency. Studer et al. [65] indicated that the enzymatic hydrolysis efficiency of untreated biomass was not correlated with the S/G ratio of lignin. Therefore, the consistency of the conclusion on the effect of the S/G ratio on enzymatic hydrolysis of lignocellulose remains to be determined.

The relative abundance of G, S, and H has been suggested to affect the enzymatic hydrolysis efficiency. Kim et al. [73] found that oxalic acid pretreated biomass was more pronounced to inhibit the enzymatic hydrolysis compared to sulfuric acid pretreated biomass, the reason for this is that the former contained a large amount of G unit lignin which had a high affinity for enzymes. Ko et al. [74] also found that softwood lignin-containing a higher G unit had a stronger inhibition effect on enzymatic hydrolysis than hardwood lignin. Furthermore, the lignin extracted from woods has a more significant inhibition effect on enzymatic hydrolysis than the one extracted from herbaceous in which the lignin has a loose structure and a high H unit content [75]. Genetic engineering to change the three monomers’ relative abundance is an alternative method to reduce cell wall structural recalcitrance and improve enzymatic hydrolysis efficiency and is especially effective in increasing the S monomer content to achieve a high hydrolysis yield [76, 77]. This might result from the higher affinity of the G unit for enzymes than the S unit [74, 78]. Fu et al. [79] generated the switchgrass mutants with a reduced S/G ratio and the ethanol yield was increased by 38% compared to the native plants. Bonawitz et al. [80] created a new mutant of Arabidopsis whose lignin is almost entirely composed of H units, and saccharification efficiency was significantly increased.

The presence of hydroxycinnamates in lignin was found to affect the biomass recalcitrance. Ferulates (FA) and *p*-coumarates (*p*CA) are two important hydroxycinnamates in grasses [81]. It was reported that the presence of FA makes the lignin close to the polysaccharide, thus increasing the recalcitrance of the substrate [82]. Reinoso et al. [83] suggested that the pretreated substrate was resistant to enzymatic hydrolysis due to the presence of ester-linked *p*CA. The cleavage and/or modification of FA and *p*CA through some alkaline pretreatments could improve biomass digestibility. Yoo et al. [84] reported that ammonia pretreatment could crack FA cross-linkages and promote lignin depolymerization, thus improving the digestibility of biomass. Martínez et al. [85] found that NaOH pretreated simultaneously removed the xylan and *p*CA in sugarcane bagasse, thus improving the cellulose accessibility thereof saccharification efficiency.

The linkages type of lignin in pretreatment has a significant impact on enzymatic hydrolysis of lignocellulose. The pretreatment results in the cleavage of original linkages and the formation of new linkages in lignin, thus changing the lignin properties. The cleavage of β-*O*-4 linkages can facilitate the disruption of cell wall matrix. For example, considerable β-*O*-4 linkages were broken in the autohydrolysis pretreatment of poplar [86] and the dilute acid pretreatment of switchgrass [69]. Lai et al. [87] prepared extractable lignin (EL) and milled wood lignin (MWL) isolated from ethanol organosolv pretreated wood sawdust. It was found that EL showed a lower inhibitory effect on enzymatic hydrolysis than MWL, due to its lower abundance of β-5 linkages. Jeong et al. [88] obtained LCC from raw materials and Fenton oxidation–hydrothermal pretreatment of larch and yellow poplar and investigated their structural characteristics. They found that the contents of β–β and β-5 in pretreated larch LCC were higher than those in pretreated yellow poplar LCC. Correspondingly, enzymatic hydrolysis efficiencies of pretreated yellow poplar and larch were 93.53% and 26.23%, respectively. These findings indicated that the presence of β–β and β-5 linkages in pretreated biomass was not conductive to enzymatic hydrolysis. However, due to the generation of more free phenolic hydroxyl, the cleavage of the β-*O*-4 linkages of lignin during the pretreatment may be detrimental to enzymatic hydrolysis. Yoo et al. [89] found that the content of β-*O*-4 linkages of residual lignin reduced significantly during the organosolv pretreatment of poplar. The consumption of β-*O*-4 linkages resulted in an increase in the phenolic hydroxyl content of lignin in pretreated poplar, thus increasing the adsorption capacity of cellulase onto the lignin fraction. In addition, the condensation of lignin through the formation of new carbon–carbon bonds generally occurred in the pretreatment step, leading to an increase in the hydrophobic interaction between the enzyme and lignin [90]. Song et al. [91] found that DES pretreatment of willow and corn stover caused their lignin fragments to undergo condensation reactions. The recondensation of lignin increased the hydrophobic interactions between lignin and cellulase, thus promoting the adsorption of cellulase onto lignin.

Lignin forms the LCC linkages with side chains of hemicellulose by covalent bonds, resulting in the lignocellulose recalcitrance [92]. Min et al. [93] found that the presence of LCC reduced enzyme accessibility to the substrate and inhibited enzymatic hydrolysis efficiency. Meanwhile, they also found that the LCC content was proportional to the S to vanillin (V) ratio for a similar levels of lignin contents, and the higher the S/V ratio, the more obvious the inhibitory effect on enzymatic hydrolysis. Singh et al. [94] suggested that ammonia fiber explosion pretreatment improved enzymatic hydrolysis efficiency by breaking the LCC linkage between lignin and carbohydrate. Li et al. [95] indicated that the cleavage of LCC bonds by hydrothermal pretreatment is beneficial to improve the digestibility of cellulose, because LCC can contribute to the biomass recalcitrance.

Moreover, functional groups (methoxyl, phenolic hydroxyl, aliphatic hydroxyl, and carboxyl) of lignin affect the efficiency of enzymatic hydrolysis by altering the interactions between lignin and enzymes. Huang et al. [90] found that the phenolic hydroxyl group was associated positively with the unproductive adsorption of the cellulase on lignin, but negatively with the aliphatic hydroxyl group. Yu et al. [96] also investigated the effect of the hydroxyl groups on enzymatic hydrolysis using MWL and found that the cellulase adsorption on MWL was increased with the decrease of total hydroxyl. The decrease in aliphatic hydroxyl is the main reason for the change of total hydroxyl. A similar study also observed that lignin with high phenolic hydroxyl group content shows strong non-productive adsorption of cellulase [97]. The reason for these phenomena is that the cellulase–lignin interaction was changed due to the formation of hydrogen bonds. Yang and Pan [98] found that the inhibition effect of lignin was reduced by hydroxypropylation reaction to block the free phenolic hydroxyl group. Carboxyl groups affect the adsorption capacity between lignin and enzyme by increasing the hydrophilicity and the negative charge of lignin. Ying et al. [99] found that alkali pretreatment combined with carboxylation post-treatment could increase the enzymatic efficiency by 1.5–3.4 times, which was attributed to the increase in the carboxylic group content of lignin after post-pretreatment. Guo et al. [100] found that MWL from corn stover had a stronger affinity with cellulase, due to its lower carboxyl content and higher phenolic hydroxyl content compared to lignin from kenaf and aspen. In addition, with regard to the effect of methoxyl on enzymatic hydrolysis, no correlation was found between methoxyl and enzymatic hydrolysis efficiency [101]. Qin et al. [102] investigated the relationship between methoxyl and enzymatic hydrolysis efficiency using lignin model compounds, and found that additional methoxyl had a positive or negative effect on enzymatic hydrolysis, which was related to the type of lignin model compound. The inconsistent correlation between functional groups and enzymatic hydrolysis may be related to biomass sources and pretreatment methods. In the future, a clearer impact of functional groups on enzymatic hydrolysis needs to be found.

The characteristics of lignin, including the content, distribution and structure, have an important influence on the enzymatic hydrolysis of lignocellulose. The pretreatment partially eliminates the negative effect of lignin on enzymatic hydrolysis by reducing the content of lignin [30]. The structure changes of lignocellulose caused by the redistribution of lignin can reduce the recalcitrance of lignocellulose and increase the accessibility of the enzyme to cellulose [52]. In addition, residual lignin with high S/G ratio and high carboxyl and aliphatic hydroxyl content is conducive to the enzymatic hydrolysis process. However, high contents of phenolic hydroxyl, hydroxycinnamates, β–β, β-5, and LCC linkages are detrimental to the enzymatic hydrolysis process [65, 81, 88, 100].

## Effect of introduced lignin on enzymatic hydrolysis

### Water-insoluble lignin (WIL) derived from different sources of biomass feedstock

Various origins of lignin have been used to clarify the inhibitory effect on enzymatic hydrolysis, which is generally extracted from raw material or pretreated biomass using chemical solvent and recovered by precipitation. Lignin recovered under neutral or acidic conditions are mostly insoluble in water. The sources and extraction methods of lignin have an important impact on lignin chemistry and enzymatic hydrolysis efficiency. Most studies show that lignin can inhibit cellulose hydrolysis, and the inhibitory effect was related to the source of lignin, as reported by Zhang et al. [103] and Pan et al. [101]. Different from the conclusion that most lignins inhibit enzymatic hydrolysis, some lignin have little influence on the hydrolysis of pretreated biomass. Interestingly, several recent reports also suggested that some technical lignin have a boosting effect on enzymatic hydrolysis. For example, it was determined that the addition of ethanol organic solvent lignin significantly improved enzymatic hydrolysis efficiency [104, 105]. This may be because the polarities of ethanol and water are similar, and the ethanol organosolv lignin has a low Mw and allows itself partly water-soluble. Therefore, the enhanced hydrolysis conversion is caused by this part of the WSL. Moreover, an increase in hydrolysis yield can be achieved by increasing carboxyl content in isolated lignin preparations [106]. Jia et al. [107] investigated the effect of two extraneous lignin additives on the enzymatic hydrolysis of cellulose. The two lignin samples were taken from the γ-valerolactone/water pretreatment of corn stover. After, respectively, adding 2 g/L of two lignins, the glucan conversion of cellulose was increased from 28.0% to 37.4% and 31.3%. Hassanpour et al. [108] prepared the acid–glycerol (AG) pretreated sugarcane bagasse and dilute acid pretreated sugarcane bagasse and recovered the lignin from enzymatic hydrolysis residual of AG pretreated sugarcane bagasse. After adding lignin produced from AG pretreated process to the two substrates, lignin did not inhibit the enzymatic hydrolysis efficiency of AG pretreated sugarcane bagasse, but increased the enzymatic hydrolysis efficiency of dilute acid pretreated sugarcane bagasse from 33 to 38%. In addition, Lan et al. [109] suggested that the addition of sugarcane bagasse lignin that recovered from the *p*-toluenesulfonic acid pretreatment hydrolysate reduced the adsorption of cellulase to sugarcane bagasse residue. While interestingly, due to the simultaneous decrease of the non-productive adsorption of cellulase onto lignin, the total enzymatic hydrolysis efficiency did not showed a detectable change. Wang et al. [110] found that the alkali lignin from liquid hydrothermal pretreated biomass had a little negative effect on the efficiency of enzymatic hydrolysis. Zhang et al. [103] also showed that ethanol lignin did not exhibit negative impact on cellulose hydrolysis, and even increased 5% glucose yield when increasing the lignin content to 40%. The effect of different WIL on enzymatic hydrolysis efficiency is summarized in Table 2.

**Table 2:** Inhibition effects of different WIL on enzymatic hydrolysis

Lignin type	Enzyme	Substrate	Enzymatic hydrolysis	Hydrolysis yield	Refs.
EOL-LP, EOL aspen and KPL	Cellic CTec 2	DASG	pH = 4.8, 50 °C, 150 rpm, and 72 h	Decreased from 27.0 to 20.0%	[66]
EOL-LP	Novozyme 22C	OPSG and OPLP	2% (w/v), pH = 4.8, 50 °C, 150 rpm and 72 h	Decreased from 49.3% and 41.2% to 42.0% and 38.1%	[104]
EOL-LP	Celluclast and Novozyme 188	Avicel	2% (w/v), pH = 4.8, 50 °C, 150 rpm and 48 h	Decreased by 67.8–86.2%	[98]
HP	Celluclast and Novozyme 188	Avicel	2% (w/v), pH = 4.8, 50 °C, 150 rpm and 48 h	Decreased by 23.0–76.0%	[98]
HEL, KL and SEL	Celluclast and Novozyme 188	Avicel	2% (w/v), pH = 4.8, 45 °C and 200 rpm	Decreased by 10.0%, 13.0% and 23.0%, respectively	[101]
PTL and CEL from steam pretreated poplar	Spezyme CP and Novozyme 188	Avicel	5% (w/v), pH = 4.8, 50 °C, 150 rpm and 48 h	Decreased by 8.6% and 11.0%, respectively	[75]
PTL and CEL from OPLP	Spezyme CP and Novozyme 188	Avicel	5% (w/v), pH = 4.8, 50 °C, 150 rpm and 48 h	Decreased by 23.0% and 25.0%, respectively	[75]
EL from OPSG	Novozym 22C	OPSG	2% (w/v), pH = 4.8, 50 °C, 150 rpm and 72 h	Improved from 72.6 to 77.7%	[105]
EL from OPSG	Novozym 22C	OPSG and OPLP	2% (w/v), pH = 4.8, 50 °C, 150 rpm and 72 h	Increased from 49.3 to 68.6% and from 41.2 to 60.8%,	[104]
Liquid hot water treated alkali lignin	Commercial cellulase	filter paper and lignin mixed	5% (w/v), pH = 4.8, 50 °C, 150 rpm and 72 h	Little inhibition effect	[110]
EL from OPSG	Cellic CTec 2	Cellulose	2% (w/v), pH = 4.8, 50 °C, 180 rpm and 72 h	No inhibition effect	[103]

*EL* extractable lignin, *EOL-LP* organosolv ethanol lignin from Lodgepole pine, *EOL aspen* ethanol organosolv lignins from aspen, *KPL* kraft pine lignin, *HP* organosolv ethanol lignin from hybrid poplar, *HEL* hardwood ethanol organosolv lignin from Lignol, *KL* kraft lignin from Aldrich, *SEL* softwood ethanol organosolv lignin from lignol, *PTL* protease treated lignin, *CEL* cellulolytic enzyme lignin, *OPSG* organosolv pretreated sweetgum, *OPLP* organosolv pretreated lodgepole pine, *GLMP* Green liquor pretreated masson pine, *MP* Masson pine, *DASG* Dilute acid pretreatment switchgrass

The Mw of introduced lignin significantly influence the efficiency of enzymatic hydrolysis [111]. Li et al. [66] found that the deposition of high Mw lignin onto fiber surface is easy, and the deposited lignin further increased the steric hindrance. On the contrary, lower Mw lignin reduced the non-productive adsorption of enzyme onto lignin. During the autohydrolysis pretreatment of spruce, the addition of 2-naphthol and dimethylphloroglucinol significantly reduced the Mw of lignin and increased the enzymatic hydrolysis efficiency of the substrate by 39% and 42%, respectively [112]. In addition, Lai et al. [105, 113] also observed that the addition of the organosolv lignin with low Mw, which obtained from the organosolv pretreatment of biomass, facilitated the enzymatic conversion of pretreated lignocellulose. In general, low Mw lignin has a less inhibitory effect on enzymatic hydrolysis than high Mw lignin, and may even have a promoting effect. However, a few case showed that higher Mw lignin may be also beneficial for the enzymatic hydrolysis of pretreated biomass. For instance, Li et al. [17] found that maximum enhancement of the enzymatic hydrolysis of DA-pretreated rice straw and alkali-pretreated rice straw of 29% and 20% occurred for adding alkali lignin with higher Mw. In short, the influence of lignin on the enzymatic hydrolysis of lignocellulose is complicated. Further study on this topic is necessary and urgent.

To gain insight into the relationship between non-productive adsorption and enzymatic hydrolysis, Langmuir model of adsorption isotherms has been adopted to measure the parameters for the adsorption of enzyme to different WILs, including the maximum adsorption capacity (*P*_ad, m_), affinity (*K*_P_) and bind strength (*P*_ad, m_ × *K*_P_). Nakagame et al. [114] found that the adsorption data of different source WILs are in good agreement with the Langmuir model of adsorption isotherm, with *K*_P_ and *P*_ad, m_ ranging from 0.48 to 4.19 L/g protein and 32.03 to 81.99 mg/g lignin, respectively. The MWL from GL-SW and GL-HW absorbed the 68% and 40% cellulase and decreased the total sugar conversion by 4% and 13%, respectively [96]. The hydrophilic modification of ethanol organosolv lignin resulted in the reduction of binding strength to cellulase from 3274.2 mL/g lignin to 73.7, 44.4, 415.4 and 373.9 mL/g lignin, thereby eliminating 76–96% of lignin inhibition [98]. The ethanol organosolv lignin from sweetgum (EOL-SG) improved the enzymatic saccharification of organosolv pretreated loblolly pine by 38.8%, while the organosolv ethanol lignin from lodgepole pine (EOL-LP) decreased the enzymatic saccharification of organosolv pretreated loblolly pine. Furthermore, the results from cellulase adsorption experiments indicated that the addition of EOL-SG decreased the cellulase adsorption to residual lignin from 61.1% to 33.2%, while the addition of EOL-LP increased the cellulase adsorption to residual lignin from 61.1% to 77.7%, which agreed with the effect of EOL-SG and EOL-LP on the enzymatic saccharification [115]. The dilute acid pretreated ERL (enzymatic residual lignin) was reported to hamper the cellulose hydrolysis, and the binding strength between dilute acid pretreated ERL and cellulase (261.5–410.9 mL/g) is higher than untreated ERL (236.0 mL/g) [116]. These results clearly demonstrated that the enzyme adsorption properties were closely related to the enzymatic hydrolysis. In recent years, quartz crystal microbalance with dissipation (QCM-D) has been used to monitor the enzyme adsorption on isolated lignin in real-time. Rahikainen et al. [117] used QCM-D to study the enzyme binding with lignin, and observed that cellulase adsorption amounts were higher for steam explosion pretreated lignin compared to untreated lignin, and therefore, the former showed the stronger inhibition effect on hydrolysis reaction. Lai et al. [87] used QCM-D to calculate the cellulase adsorption parameter, and observed the EL has a lower maximum adsorption capacity (152.63–168.09 ng/cm^2^) compared to MWL (196.71–224.73 ng/cm^2^), further the irreversible adsorption of EL (75.40 ng/cm^2^) is lower than MWL (137.35 ng/cm^2^), which is similar to increased enzymatic hydrolysis rate due to the presence of EL. The adsorption experiment with QCM-D found that fungal monocomponent enzymes (Cel6A and Cel7B) showed the high adsorption capacity and non-productive adsorption towards the steam pretreated spruce lignin, with hydrolysis yield decreasing by 66% and 60%, respectively [118].

### Water-soluble lignin (WSL) derived from sulfite pretreatment liquor or modification/fractionation of original lignin

Contrary to the traditional view that lignin always plays a negative role in enzymatic hydrolysis, some recent studies have found that the addition of WSL increased the efficiency of enzymatic hydrolysis, as shown in Table 3. Currently, the WSL that promotes the enzymatic hydrolysis includes sulfonated lignin (SL), lignosulfonate (LS) and some low Mw lignin, such as kraft lignin and alkali lignin.

**Table 3:** Inhibition effects of different WSL on enzymatic hydrolysis

Lignin type	Enzyme	Substrate	Enzymatic hydrolysis	Hydrolysis yield	Refs.
Commercial sodium LS from poplar	Cellic CTec 2	Whatman filter paper	2% (w/v), pH = 4.8, 50 °C, 200 rpm and 72 h	Improved by 30.0% to 46.0% with low Mw fraction	[129]
LS from hydrolysate of sulfite pretreated lodgepople pine	Celluclast and Novozyme 188	Lodgepople pine	2% (w/v), pH = 5.5, 50 °C, 200 rpm and 72 h	Improved from 51.0 to 90.0%	[119]
Reax 85A	Cellic CTec 2	Acid bisulfite pretreated poplar	2% (w/v), pH = 5.4, 50 °C, 180 rpm and 48 h	Improved from 76.4 to 83.5%	[120]
Commercial sodium LS	Cellic CTec 2	Kraft pulping produced lodgepole pine	2% (w/v), pH = 4.8, 50 °C, 200 rpm and 72 h	Improved from 33.0 to 55.0%	[121]
LS from sulfite pretreated poplar	Cellulase mixture	Sulfite pretreated poplar	2% (w/v), pH = 4.8 or 5.2, 50 °C, 200 rpm and 96 h	Improved by 5% to 9%, respectively, at pH 4.8 and pH 5.2	[130]
LS from poplar sulfite pulping	Cellic CTec 2	A mixture of the cellulose	2% (w/v), pH = 4.8, 50 °C, 200 rpm and 72 h	Improved from 52.0 to 67.0%	[123]
Water-soluble alkaline lignin	Cellic CTec 2	Sodium hydroxide pretreated wheat straw	2% (w/v), pH = 4.8, 50 °C, 180 rpm and 72 h	Improved from 66.8 to 76.9%	[122]
Reax 85A (sulfomethylated softwood kraft lignin)	Cellic CTec 2	GLMP	5% (w/v), pH = 4.8, 50 °C, 180 rpm and 96 h	Improved from 42.0 to 75.0%	[16]

SL was obtained by modification of alkali lignin. It has been widely reported that the addition of LS increased the efficiency of enzymatic hydrolysis and reduced the non-productive adsorption of cellulase onto lignin [16]. Moreover, the enhanced hydrolysis efficiency is associated with buffer solution pH. With increasing pH to 5.5, LS increases the enzymatic digestibility of dilute acid pretreated and sulfite pretreated substrates by 10% and 20%, respectively [119]. Similar behavior was also observed that for acid bisulfite pretreated polar, and the highest total sugar conversion achieved the 83.5% with SL addition, while the corresponding buffer solution pH was 5.4 instead of 4.8 [120]. LS can be prepared from the sulfite pulping black liquor. Zhou et al. [121] found that LS improved the hydrolysis yield of three different types of lignocellulose substrates, and noticed that low Mw LS fractions were more effective to enhance the enzymatic hydrolysis efficiency than high Mw LS fractions. The addition of LS to pretreated lignocellulose containing hydrophobic lignin led to a higher enhancement of enzymatic saccharification than those of adding LS to pretreated lignocellulose containing relatively hydrophilic lignin. Similar studies also found that LS was beneficial to enzymatic hydrolysis of acidic bisulfite pretreated and green liquor pretreated poplar, but had no effect on sulfite–formaldehyde pretreated poplar [16]. These phenomena suggest that the effect of LS on enzymatic hydrolysis is related to the residual lignin, and that WSL promotes the cellulose hydrolysis most likely related to the "competitive" adsorption of WSL and residual lignin to cellulase.

In addition, soluble alkali lignin and kraft lignin also have a positive effect on enzymatic hydrolysis. Jiang et al. [122] found that soluble alkali lignin from pulping spent liquor promoted the  enzymatic hydrolysis of wheat straw, so they proposed a method to improve the hydrolysis efficiency of lignocellulose by adding soluble alkali lignin. Kraft lignin is prepared by acid deposition and contains a fraction of soluble kraft lignin. The soluble kraft lignin binds to cellulase and counteracts the inhibitory effect of insoluble kraft lignin on enzymatic hydrolysis, which also provides evidence that insoluble kraft lignin has no significant inhibitory effect on enzymatic hydrolysis [123].

Based on the inspiration that WSL can increase the efficiency of enzymatic hydrolysis, attention has been paid to the hydrophilic modification of lignin reduced the inhibitory effect of lignin on enzymatic hydrolysis [124, 125]. Sulfonation modification, oxidation modification and carboxylation modification of alkali lignin [99] can reduce the lignin contact angle by 61.0–70.0%, and correspondingly lower the unproductive adsorption of cellulase. Similarly, sulfonation and carboxylation modifications of lignin [98] also increased the hydrophilicity of lignin and greatly improved enzymatic hydrolysis efficiency of Avicel by 8.0–15.3% compared with the unmodified lignin. These conclusions also support the phenomenon that the sulfite pretreated biomass feedstock has a high enzymatic saccharification efficiency [126–128]. Another study also found that alkali lignin inhibited the efficiency of enzymatic hydrolysis, but the alkali lignin modified by PEG1000 led to relatively high efficiency of enzymatic hydrolysis [123]. In general, the hydrophilic modification of lignin could reduce the non-productive adsorption of cellulase onto lignin and improve the efficiency of enzymatic hydrolysis. This phenomenon indicates that the non-productive adsorption mediated by hydrophobic interactions is the main reason for low enzymatic saccharification efficiency. The finding that WSL improves enzymatic hydrolysis efficiency allows to add high value to lignin and enables the full-component utilization of lignocellulose.

The effect of introduced lignin on enzymatic hydrolysis has been widely studied in recent years. Most water-insoluble lignin has an inhibitory effect on enzymatic hydrolysis, and the inhibitory effect is different, depending on the pretreatment methods and the source of lignin [101]. Nevertheless, some water-insoluble lignins with lower Mw also exhibit a promoting effect on enzymatic hydrolysis [111]. In recent years, water-soluble lignin derived from sulfite pretreatment liquor or modification/fractionation of isolated lignin has been found to promote the efficiency of enzymatic hydrolysis [16]. Hydrophilic modification of introduced lignin is an effective strategy to improve the efficiency of enzymatic hydrolysis [123].

## Interaction between lignin and cellulase

**Fig. 2:**
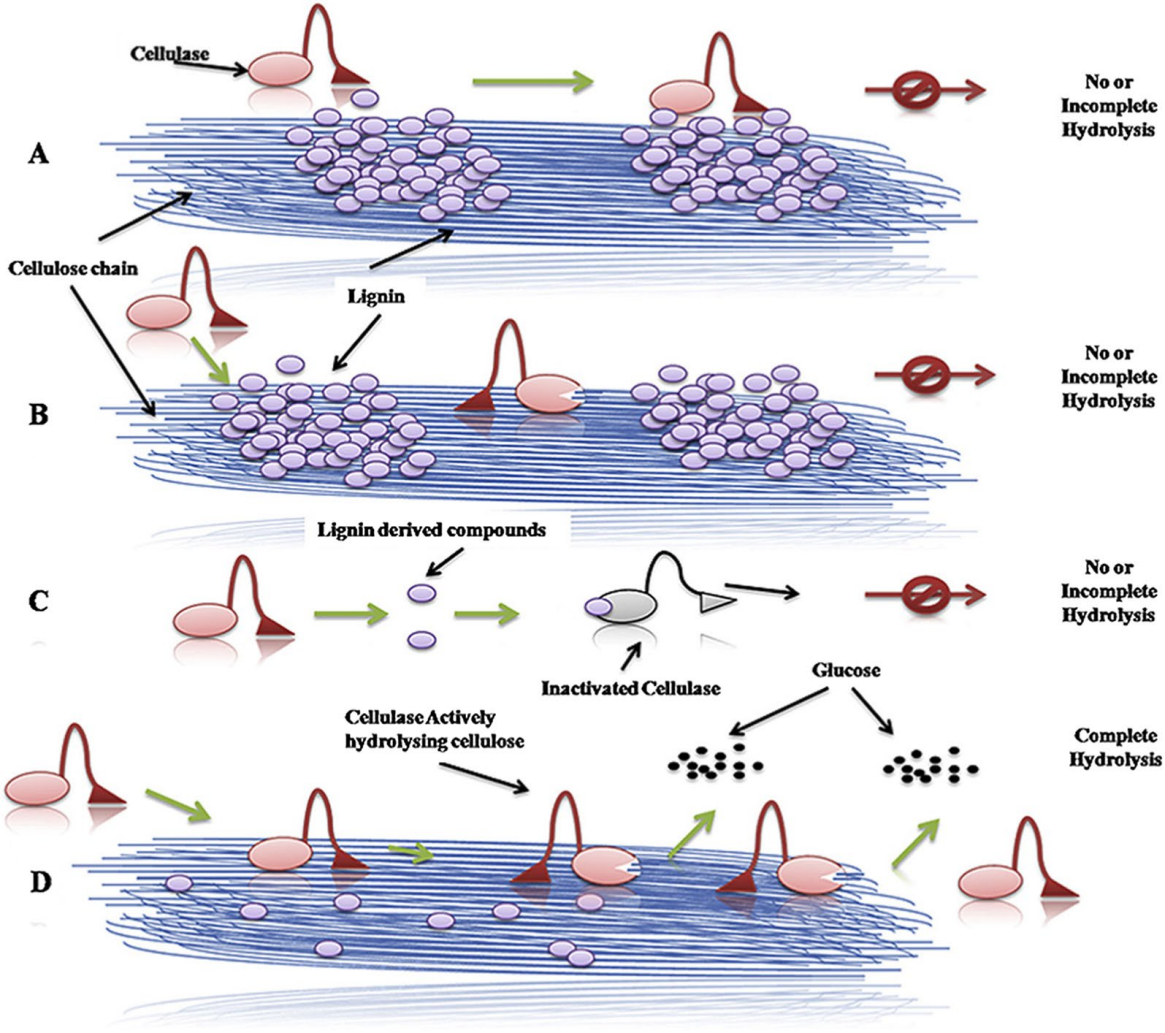
Non-productive adsorption of cellulase onto lignin (**A**), physical blockage of cellulase progress on lignocellulose chain (**B**), enzyme inhibition due to soluble lignin-derived compounds (**C**), and normal functioning of cellulase on cellulose chain to release glucose in presence of no or very low amount of lignin (**D**) [131]

The understanding of enzyme–lignin interactions is essential for reducing the inhibitory effect of lignin and enhancing the hydrolysis yield. However, due to the heterogeneity of the lignin from different sources, the mechanism of inhibition is still unclear. Currently, it is well-known that lignin hinders the efficiency of enzymatic hydrolysis in three main ways: (1) steric hindrance; (2) non-productive adsorption; (3) inhibitory effects of soluble phenols compounds (Fig. 2).

### Steric hindrance

The steric hindrance of lignin is an important factor to block the enzymatic hydrolysis except for unproductive adsorption. Vermaas et al. [132] simulated the interaction between cellulase and model biomass containing cellulose and lignin and found that lignin bound to the hydrophobic surface of cellulose, causing steric hindrance, hindering productive adsorption between enzyme and cellulose, and delaying enzymatic hydrolysis. Djajadi et al. [133] showed that hydrothermally pretreated grass biomass lignin acts as a physical barrier to hinder enzymatic degradation of cellulose, rather than induces the inhibition by non-productive adsorption of enzymes. In addition, the pretreatment of the biomass raw material also contributes to the formation of physical barrier. For example, lignin droplets and the pseudo-lignin formed during the pretreatment can block the productive binding of cellulase and cellulose [134]. Moreover, the deposition and redistribution of lignin on the cellulose surface also block the access of enzymes to cellulose [135, 136].

### Unproductive adsorption of enzyme on lignin

**Fig. 3:**
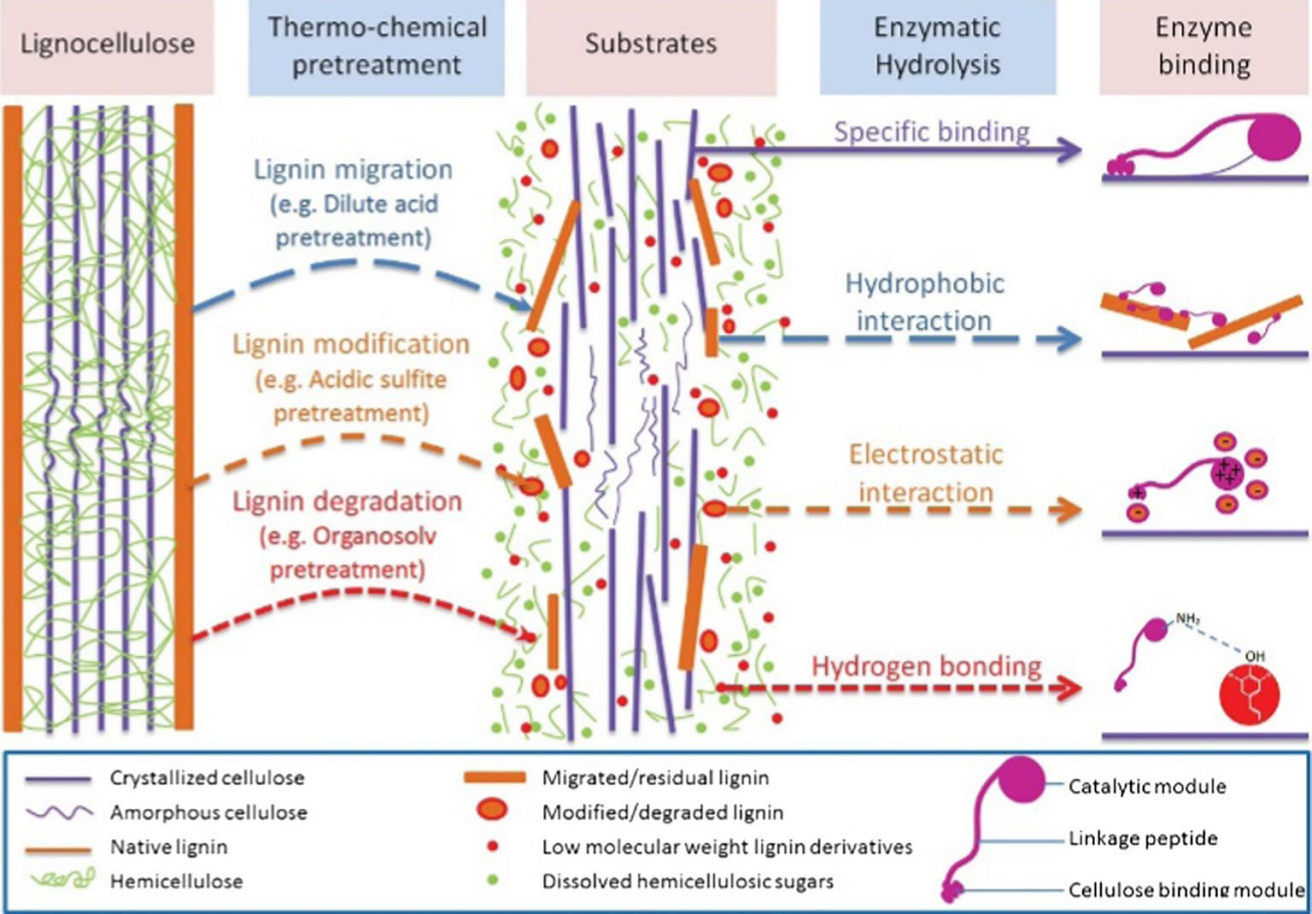
Schematic illustration of cellulase–lignin interactions dependent on lignin alteration during biomass pre-treatment [139]

Unproductive adsorption is considered to be a key mechanism for lignin to inhibit enzymatic hydrolysis (Fig. 3). Martín-Sampedro et al. [137] reported that both Cel7A and Cel7B have strong binding to cellulose and lignin, but Cel7B has a strong affinity with lignin, causing a broader effect on cellulose hydrolysis reaction. Yarbrough et al. [6] found that there is almost no β-glucosidase and endoglucanase enzyme proteins in the presence of lignin using gel electrophoresis analysis, which shows that the lignin inhibits the enzyme activity through adsorption. Qin et al. [138] indicated that the adhesion forces between kraft lignin and cellulase were higher than forces between hydroxypropyl cellulose and cellulase, which indicated that hydrophobic interaction seems to be the main attraction force for cellulase to bind lignin. The non-productive adsorption of enzyme onto lignin is categorized into three types of driving force: hydrophobic interactions, electrostatic interactions, and hydrogen bonding; whereas which one plays a dominant role depends on the complexity of the lignin structure from different sources, the type of the enzyme, and the experimental conditions. The three interactions are described below.

#### Hydrophobic interactions

Hydrophobic interaction is thought to be the main mechanism for the unproductive adsorption of enzymes and lignin [140]. After the cellulase is dispersed in water, the hydrophobic group of the cellulase stretches in the aqueous solution, which makes the hydrophobic cellulase and the solid surface adsorb together due to the hydrophobic interaction. Thus, enzymes having different hydrophobicity show different adsorption on lignocellulose substrates. Hydrophobic interaction is strongly related to the cellulose binding domain (CBM) of cellulase that is composed of specific amino acid sequences. For example, the hydrophobicity of CBM in Cel7B is higher than that of Cel7A, so that Cel7B more easily adsorbs on lignin [141]. In addition, it has been found that the inhibition effect was related to the cellulase component. Lu et al. [142] observed that lignin has the greatest reduction in Cel7A enzyme activity, followed by xylanase, then by EGs (Cel5B, Cel5C, and Cel7B) and β-glucosidase.

In addition to the hydrophobicity of cellulase, the hydrophobicity of lignocellulose as a solid substrate will also affect the adsorption of cellulase on the substrate, and the presence of lignin will increase the hydrophobicity of the substrate. The same conclusion was further verified by other researchers, when using films prepared by a spin coater to measure the hydrophobicity of different lignins (softwood sulfate lignin, softwood and hardwood MWL) and cellulose [143–145]. These studies proved that the hydrophobicity of lignin and enzymes played an important role in the non-productive adsorption of cellulase onto lignin, and were related to the reduction of lignocellulose hydrolysis efficiency.

#### Electrostatic interactions

Electrostatic interaction is also responsible for the non-productive adsorption of enzymes onto lignin. In general, the enzyme surface is ionic-charged due to the presence of carboxyl, amino and phosphate groups, etc. The net charge of enzyme molecules is related to its isoelectric point (pI), component and solution pH. For example, when the solution pH is above the pI, the cellulase is negatively charged, otherwise positively charged. Most cellulases used for enzymatic hydrolysis have pI values ≤ 4.8, showing a positively charge at the enzymatic hydrolysis reaction (pH = 4.8–5.0). In addition, the charge carried by cellulase is also related to their compositions. During solution pH of 4.8 to 5.0, Cel6A, Cel12A and β-glucosidase from *Trichoderma reesei* are positively charged, while Cel7A, the Cel7A core, Cel7B and Cel5A are negatively charged [114]. On the other hand, lignin isolated after pretreatment usually contains carboxyl, phenol hydroxyl and alcohol hydroxyl groups [146], and sulfonation-modified lignin also has sulfonic acid groups. Lignin is usually negatively charged under acidic enzymatic hydrolysis conditions (pH = 4.8–5.0), so electrostatic attraction usually occurs between cellulase and lignin. Moreover, some researchers showed that electrostatic interaction is related to the changes in pH. Baig et al. [147] observed that the adsorption of lignin and cellulase was significantly reduced in an elevated pH of 4.8 to 5.5. The decrease in the adsorption of cellulase to lignin is due to the increase in the negative charge of the cellulase at raised pH, thereby enhancing the electrostatic interaction. Lou et al. [148] showed that at an elevated pH from 4.5 to 6.0, the negativity of the zeta potential of lignin in softwood isolated by cellulolytic enzyme and protease was increased, thereby increasing the hydrophilicity of lignin and reducing its affinity towards cellulase. This study contradicts the recognized concept that the optimal pH value for enzymatic hydrolysis is 4.8–5.0. This finding has certain scientific and practical significance, since increasing the pH is easy to implement commercially at low capital costs.

#### Hydrogen bonding interactions

Hydrogen bonding interactions between cellulase and lignin have also been reported in several studies. The hydrogen bonds are thought to be formed between the hydroxyl groups in cellulose and lignin. The phenolic hydroxyl group in lignin is mainly involved in the adsorption of cellulase to lignin driven by hydrogen bonding. Due to the higher content of phenolic hydroxyl group in organosolv lignin, it is more effective than steam explosion lignin for cellulase adsorption. However, during the hydrolysis of filter paper, the addition of hydroxypropyl organosolv lignin enhances the hydrolysis yield compared to the addition of steam pretreated lignin [149]. Qin et al. [138] used atomic force microscopy (AFM) to measure the adhesion forces between lignin and enzyme and found that hydrogen bonding promoted the lignin–enzyme interaction to some extent. Zhang et al. [150] found that the addition of the surfactant such as PEG could form the hydrogen bonding with the phenolic hydroxyls of lignin, which prevented the non-productive adsorption of cellulase onto lignin through hydrogen bonding. In addition, aliphatic hydroxyl groups in lignin have been reported to be involved in hydrogen bond formation as well. However, the negative impact of lignin on the enzymatic hydrolysis of pretreated substrates may be reduced by increasing its hydrophilicity due to the presence of carboxylic acid in lignin [106]. Unfortunately, few researches have reported the effect of hydrogen bonds on the non-productive adsorption of cellulase onto lignin. Hence, to provide theoretical guidance for enzymatic hydrolysis, further experiments about the hydrogen bonding interaction still needed be conducted.

### Inhibition effect of soluble lignin-derived phenolic compound

The high pretreatment severity can result in the formation of soluble lignin-derived phenolic compounds [151]. When exposed to lignin-derived phenolic compounds, the enzyme will be immediately inactivated, causing a steady loss of enzymatic activity with the prolonged exposure to phenolic compounds [152, 153]. The inhibitory effect based on phenolic compounds is attributed to the co-precipitation with cellulase, depending on the microbial origin of cellulase, the types of enzyme and phenolic compounds [77, 154]. Kim et al. [155] carried out post-pretreatment washing on steam explosion pretreated mixed hardwood, and found that the addition of washate concentrates significantly reduced the glucose yield from 88 to only 20–30%. The inhibitory effect mainly comes from phenolic compounds of lignin (concentrations between 0.5–1.0 g/L), which are identified as gallic acid, syringic acid and guaiacol/ coniferyl alcohol. The β-glucosidase from *Trichoderma reesei* is more susceptible to inhibition by phenolic compounds than the β-glucosidase from *Aspergillus niger* [153, 156]. It has been proven that supplementing *Trichoderma reesei* cellulase with a high β-glucosidase enzyme formulation from *Aspergillus niger* can effectively enhance overall cellulase activity. At present, some strategies have been used to alleviate the inhibition effect of phenolic compounds. Peciulyte et al. [157] reported that in commercial enzyme preparations modified by the addition of lytic polysaccharide monooxygenases, phenolic compounds are actually beneficial, because these enzymes need reduce the equivalents to function. The study found that selecting the Miscanthus grass and wheat straw with less recalcitrance feedstocks can make hydrothermal pretreatment perform under mild conditions, which effectively reduces the release of phenolic compounds [158].

### Mechanisms of interactions on WSL and cellulase

**Fig. 4:**
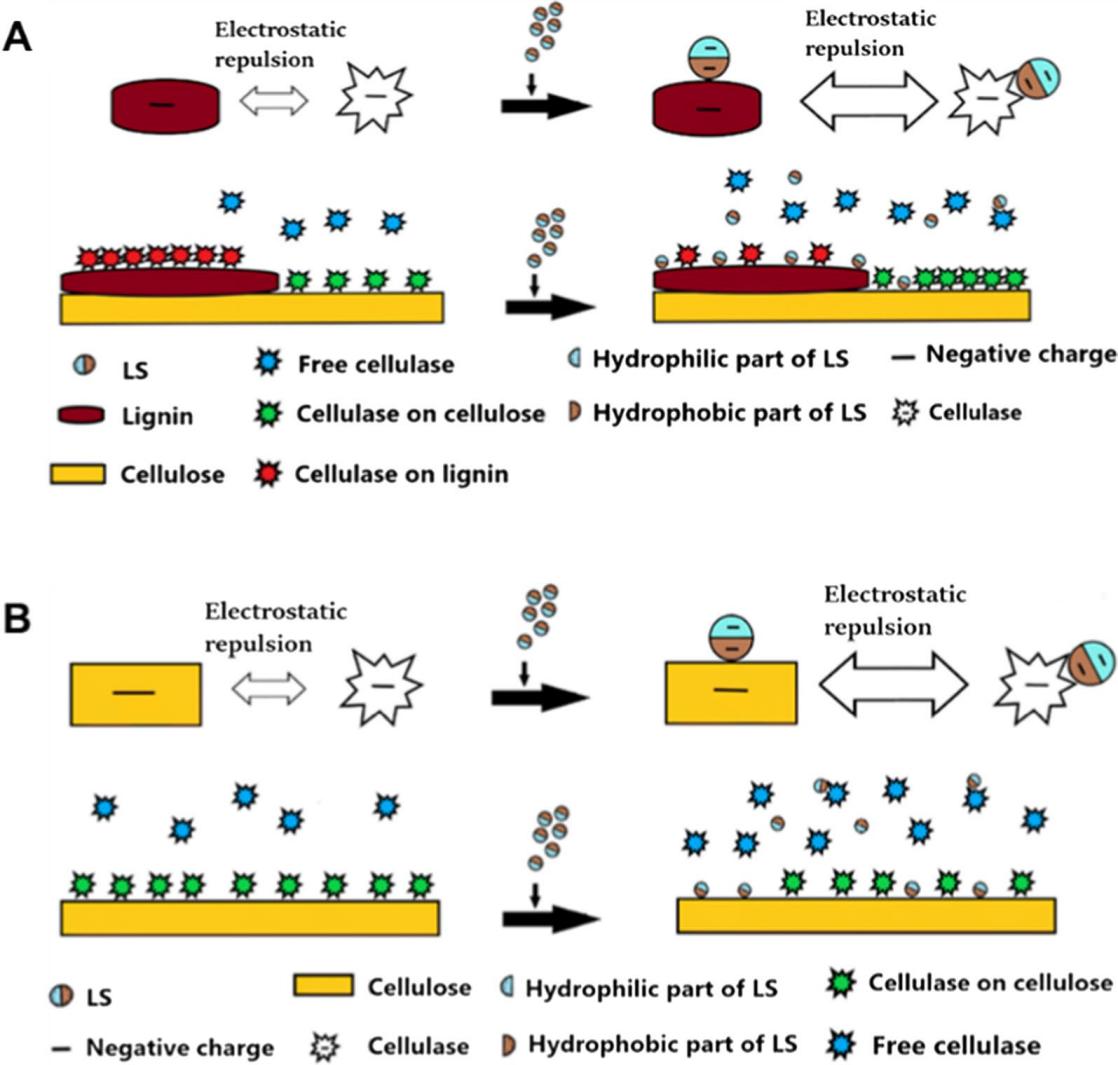
Schematic diagram of cellulase adsorption on lignocellulose (DA-SCB) before and after adding LS (**A**) and schematic diagram of cellulase adsorption on pure cellulose (Avicel) before and after adding LS (**B**) [160]

WSL, as a novel additive, is widely believed to decrease non-productive adsorption of cellulase onto lignin and promote enzymatic hydrolysis. Established data showed that the promoting effect of WSL on enzymatic hydrolysis could be related to the lignin–cellulase complex formed by the binding of WSL and cellulase, which is the key mechanism for WSL to promote enzymatic hydrolysis. Previous research indicated that LS contains the same ionizable groups as other anionic polyelectrolytes, which can bind to BSA with the formation of LS–BSA complex [159]. Similarly, Wang et al. [130] reported that LS could bind with cellulase to form a hydrophilic cellulase–LS complex, making the cellulase a more negative charge than the original. Therefore, although LS is a lignin derivative, it reduces the interaction between enzyme and lignin by weakening hydrophobic and electrostatic interactions. Zheng et al. [160] reported that the addition of LS led to the formation of LS–DA–SCB (dilute acid pretreated sugarcane bagasse) lignin complexes and LS–cellulase complexes, which increases the electrostatic repulsion between cellulase and DA-SCB lignin and results in the increased binding site of cellulase to cellulose, thereby reducing the non-productive adsorption of cellulase onto DA-SCB lignin (Fig. 4A). LS, as a surfactant can reduce the non-productive adsorption of enzymes onto lignin. Leu and Zhu [161] showed that LS can function as a surfactant to reduce non-productive adsorption and increase enzyme activity. Zhou et al. [121] also speculated that LS, with a low affinity for cellulase due to its hydrophilic surface, acts as a surfactant to block the site of residual lignin on cellulase, thus reducing the non-productive binding of cellulase onto lignin and improving the enzymatic saccharification of lignocellulose. In addition to LS, lignin–PEG (the modified isolated lignin) has been developed to reduce the non-productive of cellulase onto lignin by dispersing cellulase aggregates [162].

LS has been extensively believed to hinder the pure cellulose enzymatic hydrolysis. Zheng et al. [160] showed that LS could bind to Avicel and form the Avicel–LS complexes, which occupied the binding site of cellulase and Avicel, thus lowering the productive adsorption of cellulase on Avicel (Fig. 4B). This finding was also confirmed in the studies of Wang et al. [119] and Liu et al. [163]. They suggested that the non-productive adsorption of cellulase onto LS is the main reason for the inhibition. Nevertheless, few researchers have also found that LS could promote the hydrolysis of pure cellulose. Lou et al. [123, 129] observed that adding three commercial sodium lignosulfonates with different Mw could improve the enzymatic hydrolysis efficiency of pure cellulose. The authors thought that the LS–cellulase complex could improve and stabilize the binding of cellulase and cellulose. Zhou et al. [121] found that low Mw SL promotes the efficiency of enzymatic hydrolysis of pure cellulose. The mechanisms of WSL promoting enzymatic hydrolysis of lignin-containing biomass are mainly categorized into two types: (1) The addition of WSL leads to competitive adsorption of WSL with residual lignin, forming the WSL–cellulase complexes; (2) The WSL acts as a surfactant to stabilize the cellulase and increase the accessibility of enzyme to cellulose [130, 161]. However, most WSL has an inhibitory effect on pure cellulose substrates. This inhibition effect is attributed to the non-productive adsorption of cellulase onto WSL, thereby reducing the avaliable adsorption sites of cellulase on cellulose [163]. Overall, although it has been extensively reported that the formation of WSL–cellulase complexes can promote the enzymatic hydrolysis of lignin-containing substrates, it is not yet fully understood how complexes impacts the lignin–enzyme interaction, and thus the effect mechanism of WSL on lignocellulose still needs to be further explored.

### The newly advanced technology to elucidate lignin–enzyme interactions

In the past few years, various advanced analysis techniques have been used to elucidate the interaction between lignin and cellulase, including quartz crystal microbalance with dissipation (QCM-D), surface plasmon resonance (SPR), atomic force microscopy (AFM), Fourier transform attenuated total reflection infrared spectroscopy (FTIR-ATR), nuclear magnetic resonance (NMR) spectroscopy, fluorescence spectroscopy (FLS), and molecular dynamics (MD) simulations. Among them, QCM-D, SPR, and AFM have made greater progress in clarifying the interaction between lignin and cellulase.

QCM-D was widely used to observe the adsorption/desorption and kinetics of cellulase on the cellulose/lignin thin film in real time. He et al. [164] applied QCM-D to study the effect of pseudo-lignin and residual lignin on enzymatic hydrolysis. The dynamics of enzymatic hydrolysis showed that when pseudo-lignin and residual lignin were mixed with cellulose, the maximum glucose release was reduced and the time to reach the maximum enzymatic hydrolysis efficiency was longer than that of pure cellulose. Jiang et al. [165] prepared a full-component lignocellulose ultrathin film using the LiCl/DMSO solvent system. The in situ enzymatic hydrolysis of the film in QCM-D verified the non-productive adsorption of cellulase onto lignin.

SPR is another technology to explore lignin–enzyme interactions by simulating adsorption–desorption model. It is an optical technology that can be used to detect the thickness and structure changes of the ultrathin adsorption layer on the metal surface [166]. SPR could been used to study the kinetic parameters of the adsorption of cellulase onto lignin films during the enzymatic hydrolysis process [167]. Pereira et al. [168] used SPR to conduct the enzyme adsorption experiments in buffer solutions with different ionic strengths. The authors found that electrostatic interaction plays an important role in the adsorption of various types of lignin to cellulase. The mechanism of amphiphilic lignin derivatives promoting enzymatic saccharification was also studied using SPR [169].

Using AFM can directly measure specific interaction forces between lignin and cellulase and obtain surface images. Furthermore, AFM probes can be modified to have specific chemical characteristics, allowing AFM to measure the interaction between specific materials and molecules. Arslan et al. [170] used AFM to measure the nanoscale steric forces between a model surface with hydrophobic residues of cellulase and biomass substrates pretreated by different methods. The results indicated that organosolv pretreatment should be the first choice to reduce the non-productive adsorption of enzymes onto lignin. AFM has been also used to measure the nanoscale forces between the carbohydrate binding module (CBM) of Cel7A and three lignocellulosic substrates. The results indicated that the overall adhesion forces of substrates to CBM were proportional to the surface lignin coverage. The authors also suggested that changing the surface hydrophobicity and surface energy of lignin is a prerequisite to avoid the non-productive adsorption of cellulase onto kraft lignin [171].

## Conclusions and prospects

Enzymatic hydrolysis of biomass is a key step for lignocellulose bioethanol production via sugar platform. However, lignin endows the biomass recalcitrance of lignocellulose and irreversibly adsorbs cellulase, thus reducing the hydrolysis efficiency. The lignin content, distribution and structural characteristics in substrate have been found to influence the non-productive adsorption of cellulase onto lignin. In addition, due to the complex structure of lignin, the inhibitory effect of introduced water-insoluble lignin on enzymatic hydrolysis varies with the sources of lignin. Interestingly, adding water soluble lignin to the hydrolysis system can reduce the non-productive adsorption of cellulase onto lignin and promote hydrolysis. The hydrophobic, hydrogen bonding and electrostatic interactions are the main causes of the non-productive adsorption of cellulase onto lignin. The formation of the WSL–cellulase complexes is crucial to improve the enzymatic hydrolysis efficiency of pretreated substrates. In recent years, the development of some advanced technologies (QCM-D, SPR, and AFM) provides new insights for elucidating the lignin–enzyme interactions.

However, the removal of lignin by pretreatment is costly and adversely affect the environment. It also leads to the condensation of lignin and the formation of pseudo-lignin, reversely increasing the non-productive adsorption of cellulase onto lignin. Therefore, the selection of suitable pretreatment methods along with appropriate surface modification of lignin is an effective pathway to regulate the interaction between cellulase and lignin. Genetic engineering to change the content of lignin and regulate its biosynthesis should be more considered. Although the lignin–enzyme interaction has been extensively studied, its influence on enzymatic hydrolysis is still unclear due to the complexity of the structure of lignin and enzymes. Understanding the interactions between lignin and cellulase, such as hydrophobic interaction, hydrogen bonding and electrostatic association, is the prerequisite for improving the efficiency of enzymatic hydrolysis. Much attention should be paid to establish the relationship between the lignin molecular structure and lignin–enzyme interaction from fundamental points of view to optimize the enzymatic hydrolysis. Moreover, the hydrophobic interaction is thought to be coupled with the other two interactions to drive the non-productive adsorption of cellulase onto lignin. Therefore, how to identify hydrophobic, electrostatic, and hydrogen bond interactions between lignin and cellulase, and understand the influence of single factors on enzymatic hydrolysis is one of the challenges in the future. In addition, it is worth emphasizing that the conversion of lignin into stimulant additives, for example, lignosulfonate from sulfite pretreatment, is a cost-effective method to improve the hydrolysis yield, but the mechanism still needs be further explored. To fully unlock the lignin–enzyme interactions at the molecular level as well as the promoting effect of lignosulfonate on enzymatic hydrolysis, more state-of-the-art analytical techniques should be adopted in elucidating the lignin–enzyme interactions in future studies, and the findings are beneficial to reducing the negative effect of lignin on enzymatic hydrolysis and provide a pathway to utilize the enzymes more efficiently and cost-effectively.

## Data Availability

Not applicable.

## Abbreviations

AFEX: Ammonia fiber expansion
BSA: Bovine serum protein
FA: Ferulates
*p*CA: *p*-Coumarates
PEG: Polyethylene glycol
S/V: Syringaldehyde to vanillin ratio
MWL: Milled wood lignin
WIL: Water-insoluble lignin
QCM-D: Quartz crystal microbalance with dissipation
AFM: Atomic force microscopy
SPR: Surface plasmon resonance
WSL: Water-soluble lignin
SL: Sulfonated lignin
LS: Lignosulfonate
LCC: Lignin–carbohydrate complex
EL: Extractable lignin
Mw: Molecular weight
EOL-LP: Ethanol organosolv lignins from lodgepole pine
EOL aspen: Ethanol organosolv lignins from aspen
KPL: Kraft pine lignin
HP: Ethanol organosolv lignin from hybrid poplar
HEL: Hardwood ethanol organosolv lignin from lignol
KL: Kraft lignin from Aldrich
SEL: Softwood ethanol organosolv lignin from lignol
PTL: Protease treated lignin
CEL: Cellulolytic enzyme lignin
OPSG: Organosolv pretreated sweetgum
OPLP: Organosolv pretreated lodgepole pine
GL: Green liquor
GLMP: Green liquor pretreated masson pine
MP: Masson pine
DASG: Dilute acid pretreatment switchgrass
CBM: Cellulose binding domain
pI: Isoelectric point
DA-SCB: Dilute acid pretreated sugarcane bagasse
